# Overall Maternal Morbidity during Pregnancy Identified with the WHO-WOICE Instrument

**DOI:** 10.1155/2020/9740232

**Published:** 2020-07-17

**Authors:** Stephanie Pabon, Mary A. Parpinelli, Martha B. Narvaez, Charles M'poca Charles, Jose P. Guida, Maria F. Escobar, Jose G. Cecatti, Maria L. Costa

**Affiliations:** ^1^Department of Obstetrics and Gynecology, University of Campinas School of Medicine, Rua Alexander Fleming 101, Campinas, São Paulo 13083-891, Brazil; ^2^Department of Obstetrics and Gynecology, Icesi University and Fundacion Valle del Lili, Cali 760001, Colombia

## Abstract

**Objective:**

To evaluate the prevalence of nonsevere maternal morbidity (including overall health, domestic and sexual violence, functionality, and mental health) in women during antenatal care and further analyze factors associated with compromised mental functioning and clinical health by administration of the WHO's WOICE 2.0 instrument.

**Method:**

A cross-sectional study was conducted at a referral center in Brazil with an interview and questionnaire administered to pregnant women at 28 weeks of gestation and beyond. Data collection and management were supported by REDCAP software. A descriptive analysis was performed, and a multiple regression analysis also investigated factors associated with impairment in mental conditions, functionality, and clinical health.

**Results:**

533 women at a mean age of 28.9 years (±6.7) were included, and the majority had a partner (77.1%) and secondary education (67.7%). Exposure to violence occurred in 6.8%, and 12.7% reported substance use. Sexual satisfaction was reported by the vast majority (91.7%), although almost one-fifth were sexually abstinent. Overall, women reported very good and good health (72%), despite being told that they had a medical condition (66%). There was an overall rate of anxiety in 29.9%, depression in 39.5%, and impaired functioning in 20.4%. The perception of an abnormal clinical condition was the only factor independently associated with impaired functioning and mental health in the multiple regression model. Obesity was independently associated with clinical impairment.

**Conclusion:**

During antenatal care, pregnant women in the study reported having a high rate of anxiety, depression, impaired functioning, and substance use. These issues can affect a woman's health and should be further addressed for specific interventions and improved quality of care.

## 1. Introduction

In 2015, the World Health Organization (WHO) set the new agenda for Sustainable Development Goals [1], built on the legacy of the Millennium Development Goals [2] (2000-2015), which were not fully achieved. Among the new objectives established, the third goal is to ensure a healthy life and promote well-being for all, which includes improving maternal health and reducing maternal mortality [1].

Maternal deaths have been described as the tip of the iceberg, considering maternal morbidity as the basis [3]. It is estimated that annually thousands of women worldwide suffer from complications associated with pregnancy or the postpartum period. For each maternal death, 20 to 30 women suffer from some type of morbidity [3], although these estimates are based on nonstandard methodologies [4, 5]. Severe morbidity has been extensively studied in the past decade, with standard definitions for potentially life-threatening conditions (PLTC) and maternal near miss (MNM) issued by the WHO [6]. Nevertheless, there is growing interest in understanding morbidity in a broader sense, including nonsevere morbidity and a woman's different perspective on her own well-being.

Given the lack of standardized instruments for accurate assessment of overall and nonsevere maternal morbidities, the WHO implemented the Maternal Morbidity Working Group (MMWG) in 2012 that defined maternal morbidity as “any health condition attributed to and/or aggravated by pregnancy and childbirth that has a negative impact on a woman's well-being” [3] and created an instrument, later called WOICE, for measuring maternal morbidity, focusing on health and a woman's self-perception of well-being [4, 7, 8].

The WHO-WOICE is a questionnaire designed from a matrix [7]. Its name evokes the need to listen to women's perspectives, how they perceive their pregnancies, and the possible long-term impact not only on their lives but also on the lives of their children, family, and society [9].

The MMWG conducted a pilot study in institutions of different levels of care in three different low-income countries (Jamaica, Kenya, and Malawi), where the first version of the WOICE was applied to 750 women in the antenatal period (ANC) and 740 women in the postnatal period (PPC) [4, 7], highlighting not only clinical conditions but also the relevance of exposure to violence and mental health alterations [10].

Up to now, the prevalence of nonsevere maternal morbidity remains largely unknown, especially the conditions related to domestic violence, sexual violence, and changes in mental health, social role, and functionality. These issues may have a negative impact on women's lives. A lack of understanding of these issues may lead healthcare providers to dismiss their occurrence. As a result, this study was aimed at implementing the WHO working group (WOICE) tool in a middle-income setting in Brazil, to evaluate the whole prevalence of maternal morbidity, along with factors associated with clinical, mental, or functional impairment.

## 2. Materials and Methods

### 2.1. Study Design and Sample Size

A cross-sectional study was developed, and a questionnaire created by the WHO was used to evaluate maternal morbidity. The questionnaire includes several previously validated scales. To evaluate functionality and the ability to perform daily tasks, the WHODAS 2.0 12-item (WHO Disability Assessment Schedule 2.0-12 item) version was used. For mental health assessment, the General Anxiety Disorder test, 7-item (GAD-7) and the Patient Health Questionnaire, 9-item (PHQ-9) were used [11–13]. To measure substance use, sexual satisfaction, and exposure to violence, parts of some scores already validated were used, such as the Alcohol, Smoking and Substance Involvement Screening Test (ASSIST) and Brief Sexual Symptom Checklist for Women (BSSC-W) including some questions from a questionnaire used in the Multi-Country Study on Women's Health and Domestic Violence against Women of the WHO [14–16]. The WOICE instrument was previously used in a pilot study [4, 10].

Sample size was estimated at 500 participants for convenience, as a pilot study, considering that the WOICE instrument had not been previously published by the time data collection was planned and started. The only previous study used 250 women during ANC for each setting (3 different countries) [10].

The prevalence of abnormal functioning (20.4%) was used to estimate the statistical power of the sample, setting the level of alpha significance or type I error at 5% (alpha = 0.05), with a 95% confidence interval and sampling error of 5% (*d* = 0.05). The considered sample had a power of 82.5%.

### 2.2. Data Collection

Eligible women (pregnant from 28 weeks of gestation onwards) were selected at the antenatal care outpatient clinic at the University of Campinas maternity, a tertiary and quaternary referral hospital for over 5 million inhabitants of more than 42 cities located in the metropolitan region.

The WOICE questionnaire was applied to each participant by a trained research assistant, after the informed consent form was signed. In addition to the interview, patient medical records were reviewed to confirm inclusion criteria and clinical data. The complete procedure lasted an average of 30 minutes per case.

Data collection was performed with a tablet powered by the Android operating system. All interviewers were trained, using a detailed manual of operations. The electronic signature for informed consent was also obtained. Tablets used REDCap® software, which supported data collection, transmission, verification, checking, correction, storage, and analysis of data. Electronic equipment was protected with passwords to ensure confidentiality. Tablets worked both online and offline to simultaneously feed the database, respectively, hosted in a safe energy-protected server afterwards.

### 2.3. WOICE Tool and Data Analysis

The WOICE questionnaire was originally published in English [4]. The respective translation into Brazilian Portuguese was performed and revised by experts in the area of obstetrics and was further applied as a pilot test to measure the time of application and understanding of questions. The instrument was then adapted and modified for greater clarity (Supplement 1).

The WOICE questionnaire contains three sections:

1. Collection of social and demographic information, obstetric history, violence, sexual health and risk factors, and environment
2. Functional assessment, general symptoms, mental health, and anxiety
3. Data on physical examination and medical record review

The database was built into the REDCap® software, subsequently exported to a format compatible with the statistical package SPSS (IMB, Armonk, NY, USA) for analysis.

A descriptive analysis of sociodemographic characteristics and clinical, social, and sexual conditions of the studied population was carried out, as well as the general prevalence of validated instruments included in the WOICE questionnaire and the combination of both. The instruments were WHODAS-12 to evaluate the ability to carry out daily tasks and social responsibilities and PHQ-9 and GAD-7 scores to evaluate mental health.

For WHODAS-12, the 95^th^ percentile was considered the cutoff point to diagnose dysfunctionality, according to a previous study of a similar population conducted during the postpartum period, with a score of ≥37.4 [17]. For the GAD-7 and PHQ-9 tests, a score greater than or equal to 10 per test was required to identify anxiety and depression, respectively [12, 13].

A multiple regression analysis was also performed to evaluate factors associated with impaired conditions. Three models were proposed. The first model considered that abnormal functioning was the outcome. The second model considered that the outcome was any abnormal condition for mental health (score ≥ 10 for anxiety and depression questionnaire). The predictors tested were maternal age, marital status, education, literacy, employment, travel time to healthcare facility, parity, gestational age, BMI (≥30 kg/m^2^), overall health rating, any clinical condition, preexisting conditions, and taking any medication. The third model tested factors independently associated with impaired clinical conditions (women who answered “yes” to the question: “have you been told you have anything wrong or any medical condition?”). The predictors tested were the same used in model 1 but also impaired mental health, abnormal functioning, substance use, sexual satisfaction, and violence.

### 2.4. Ethical Considerations

This study was approved by the Local Institutional Review Board of the University of Campinas (UNICAMP) on November 17, 2017, under number: #78497817.0.0000.5404. An informed consent form was always required in women over the age of 18. Adolescents as young as 13 years of age were also included. In this subgroup, the consent form was waived, due to the content on violence. To have a copy of this document or ask the adult responsible for the adolescent to sign the paper might expose people to additional risks. Nevertheless, all included adolescents were properly informed about the research and were only interviewed after giving verbal assent.

## 3. Results

**Figure 1:**
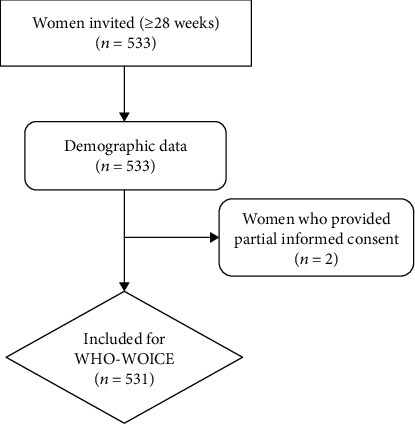
Flowchart of participant inclusion.

This study was conducted in a referral maternity unit, where 533 pregnant women were invited to participate. Of the total number of women, 531 women gave full consent and 2 did not participate in the interview but provided us with their sociodemographic data (Figure 1**).**

Among the characteristics of our population, the mean age of the women was 28.9 years (±6.7), with most women (67.0%) aged between 20 and 34 years and 10% aged less than 20 years. More than two-thirds of the women had a partner and were employed. The majority of our study population had a secondary school level, and 14.3% had a higher educational level (Table 1).

Although the study was performed in a high-risk outpatient clinic and two-thirds of the women reported being informed about having a medical condition since they became pregnant, most participants regarded their general health level as “good” or “very good” (59.4% and 12.6%, respectively) and only 6.8% regarded their health as “poor” or “very poor” (Table 2).

An interesting approach in the current study was to evaluate the number of pregnant women that had more than one type of impairment, taking into consideration all aspects of the WOICE. More than half of these women had at least one condition, and nearly one-quarter had 2 conditions. Only 2.4% had absolutely no abnormal results (Table 2).

Any substance use during the current pregnancy is also evaluated by WHO-WOICE. In this study, 12.7% of the interviewees reported substance use. Among substance users, 22.6% reported feeling an impact on their daily activities due to substance use, which was the cause of major family concern (over 60%). A very important piece of information is that more than half of the substance users had tried to stop consuming but had not succeeded (Table 3).

Women reported feeling fear or experiencing some form of physical violence by the current partner or anyone else at a rate of 6.8% (*n* = 36). This rate referred to women who had been afraid of their current/most recent husband or partner or anyone else or those that had answered positively to the question “during this pregnancy, was there ever a time when you were pushed, slapped, hit, kicked, or beaten by (any of) your husband/partner(s) or anyone else?” (Table 3).

We explored data on sexual health of interviewees and found that one-fifth of the women responded that they had stopped having sex during pregnancy. The reasons were the following: no longer having a partner (22%), medical restrictions (39.4%), and low sex drive in her partner (3.7%) or in her (34.9%). However, the remaining 91.7% of participants reported feeling sexually satisfied (Table 3).

To evaluate functionality or ability to do everyday tasks, WHODAS-12 version 2.0 found a mean score of 23.1 (±16.7) which was higher than 37.4 in 20.4% of the cases. In the GAD-7 and PHQ-9 tests for anxiety and depression, respectively, 29.9% and 39.5% of women scored 10 or more, which defined an impaired condition [12, 13] (Table 4).

**Figure 2:**
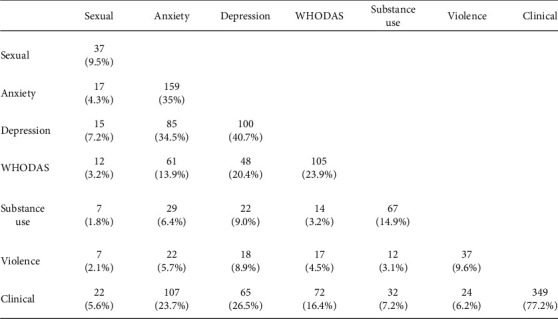
Combined social, mental, and functional impairment (antenatal care study population), *n* = 454.

The prevalence of conditions assessed by WHO-WOICE was compiled individually and in combination, as shown in Figure 2. The most frequent combined conditions were anxiety and depression (34.5%), depression and abnormal functionality (20.4%), having been informed of any clinical condition, and anxiety (23.7%) and depression (26.5%).

On multivariate analysis, a woman's clinical condition (self-report of poor health) was the factor associated with an increased risk of abnormal functioning and impaired mental health. Illiteracy was also associated with anxiety and depression. The third model for multivariate analysis considered that clinical condition (impaired clinical health) was the outcome and obesity (BMI ≥ 30) was the associated factor. In this model, substance use was protective, most likely due to characteristics of the instrument and use of a self-reported response to define health status (Table 5).

The WHO-WOICE instrument was always administered after a scheduled antenatal care visit and did not interfere with the woman's medical follow-up. Nevertheless, since questions could potentially lead to unpleasant memories and reveal exposure to violence and substance abuse, additional support was offered. We found that 35.2% of the women requested such follow-up and 94.1% sought psychological referrals (result not shown).

## 4. Discussion

This study shows the results of the WHO-WOICE version 2.0 instrument used during antenatal care at a referral maternity unit from a middle-income setting in Brazil. Overall, there was very good compliance, which shows that women are willing to participate in research during pregnancy, irrespective of whether the research is about sensitive issues such as physical or sexual violence. This may reflect an opportunity to share their experiences. Main results included a high rate of anxiety, depression, impaired functioning, and substance use.

The WHO-WOICE had once been previously employed in a pilot study, mostly in low-risk populations from low-income settings in Jamaica, Kenya, and Malawi [10]. Relative to sociodemographic data, our population was older and the majority had a partner and a much higher educational level than women in countries of the pilot study [10]. This places Brazil in an intermediate position in the obstetric transition status [18, 19]. Another marked difference in the current study was an increased rate of substance use.

It is well known that substance use leads to negative consequences during pregnancy [20–24], despite the still high prevalence of substance use in pregnant women. Some studies show that one in every three women consumes alcohol during pregnancy. Tobacco use varies between 12% and 25% and illicit drugs between 4% and 7.4%, with cannabis and cocaine being the most frequently used illicit substances during pregnancy [21, 25, 26]. It is noteworthy that an association exists between mental health disorders and substance use [27, 28]. Our results also showed a greater frequency of mental health disorders, compared with the pilot study, which could also have influenced such findings. Although the WOICE instrument does not detail the exact substance consumed by a woman, it is still an indicator that the problem should be addressed.

A surprising result was that substance use was an independent factor that may be protective of overall clinical health. A possible explanation is that the WHO-WOICE instrument considers women's perception of health. Therefore, those suffering from substance abuse may actually underreport health complaints.

We know that substance use is a public health problem, along with violence against women. It occurs especially in low- and middle-income settings and in the vicinity of big cities. The issue is of great global interest because it violates women's rights. The most common type of violence during pregnancy is psychological violence [29].

Violence against women is difficult to approach and identify. Some acts of violence are considered the norm in certain cultures. In cases identified as violence, fear is generated in both the female victim and health professionals, who refrain from reporting these cases [16, 29–31]. Our study showed that 6.8% of women had been exposed to some kind of violence, compared to 12.8% in the pilot study [10] and 8% and 11% in previous reports in urban and rural regions in Brazil, as evidenced by a multicenter study on violence against women published by the WHO in 2005 [16]. Other studies have shown a prevalence of domestic violence greater than 40% [29, 32]. It is likely that our study obtained underestimated data since women did not consider themselves to be victims of violence, despite the ill treatment [33, 34].

Another important aspect to evaluate in prenatal visits is the sexual health of pregnant women, defined by the WHO as a “state of physical, mental, and social well-being in relation to sexuality” [35]. Sexual dysfunctions include little or no interest in sex, dyspareunia, and problems such as lubrication and genital sensation that can lead to sexual dissatisfaction and subsequent sexual inactivity [36].

The literature shows that there is a decrease in sexual activity in the perinatal period, due to maternal morbidities, physiological changes, or lack of information about sexuality during pregnancy or cultural behavior [10, 36–38]. A study reported that the frequency of sexual inactivity during the first trimester of pregnancy was 24% [36], compared to our findings showing that around 20% of women stopped having sex as soon as they became pregnant. Nevertheless, most participants reported being satisfied with their sex lives, which means that some women interviewed did not need sexual intercourse to report sexual satisfaction. In pregnant women, sexual satisfaction is linked to a woman's acceptance of her body image, the type of communication with her partner, and having sex [37].

In addition, according to a systematic review and the pilot study, sexual dissatisfaction is associated with clinical or obstetrical morbidity [10, 39]. However, in our study, this was not the case. Regardless of increased overall morbidity, women mainly reported having good or very good health and sexual satisfaction. A woman's self-perception of well-being is possibly, what really matters, not the clinical diagnosis itself. Women may feel good to receive treatment and appropriate follow-up at a referral center. Another possibility is that part of the sample is just oblivious to the details and risks inherent in their conditions [40].

Another aspect evaluated by the short WHODAS 2.0 version was functionality. A previous study showed that the best cutoff point to define disability with this instrument is the 95^th^ percentile (score over 37.4 points, of a total of 100 in a sample of postpartum women). This cutoff point identified around one-fifth of included women. This was a striking result, indicating higher levels of impaired functioning than previously published [17, 40], which was in agreement with the understanding that functioning is more often impaired in the antenatal period rather than in the postnatal period.

The aim of our multivariate analysis was to identify factors associated with impaired conditions related to clinical health, overall mental, and functional health. Our results were mostly in agreement with the literature, and obesity was associated with clinical diagnosis. According to the WHO, the global prevalence of obesity increased almost threefold from 1975 to 2016 [41]. In the general population (nonpregnant), a body mass index above 30 is classified as obesity [42]. For pregnant women, there are recommendations of weight gain per gestational week depending on pregestational weight. However, in pregnancy, there is a lack of international consensus on the level that best determines obesity. Nevertheless, studies support that BMI > 30 increases comorbidity risk during pregnancy. In this study, more than half of the women had a BMI above 30.

Another relevant finding is that a clinical diagnosis may have an impact on a woman's mental health and functioning. Although this is a well-known fact, it is not commonly reported in a systematic manner. Understanding that clinical conditions may be associated with further impairment can guide interventions and improve healthcare [43].

One important point to be addressed refers to the potential clinical application of this instrument. Pregnancy is no longer understood only as a magic and beautiful situation when everything goes well. In fact, the recognition that pregnancy poses a weight for women in terms of higher morbidity directly or indirectly associated with pregnancy, plus the burden of the pregnancy itself on the woman's well-being, health and quality of life, represent a step forward in the process of listening women voices. Although relatively time consuming, the application of a questionnaire like the one assessed in the present study would allow for a full assessment of maternal morbidity in a broad spectrum. This should be performed routinely during pregnancy to properly identify, select, and manage conditions affecting the women's general and mental health, functioning and quality of life, according to their own feelings.

Our study has some limitations. The WHO-WOICE instrument evaluates a broad number of aspects. However, some are not further detailed, such as substance use (including any type of substance) and violence, with no information on previous history of violence or psychological violence. Another aspect is the duration of the questionnaire, which is a limiting factor for implementation in routine healthcare. In the future, a review of the structure of antenatal visits would be welcome to complement their features with the WOICE aspects. Furthermore, there is a lack of information on long-term follow-up or possible effects of abnormal findings using this instrument. Here, we reported that more than one-third of the women requested psychological support after talking about all the conditions included in the WHO-WOICE.

## 5. Conclusions

Maternal morbidity should be analyzed beyond severity. It has a broad spectrum that should be studied in its entirety to ensure that actions are taken for the prevention, diagnosis, and treatment of conditions other than clinical diseases. It is essential to consider women at the center of care, recognizing the existence of nonsevere morbidities. When these morbidities are routinely evaluated, a multidisciplinary approach can be used to improve healthcare. Anxiety, depression, impaired functioning, substance use, and violence are frequent conditions among pregnant women. These issues are brought to light by the use of the WHO-WOICE instrument and merit further prioritization to improve women's health.

## Figures and Tables

**Table 1:** Sociodemographic characteristics of included women (during antenatal care).

Characteristics	Variable	Total, *N* = 533	%
Maternal age	Mean (SD)	28.9 (±6.7)	
	<20 years	53	9.9
	20-34 years	357	67.0
	>34 years	123	23.1
Marital status	No partner	122	22.9
	Has partner	411	77.1
Education	Primary or less	96	18.0
	Secondary	361	67.7
	Higher	76	14.3
Literacy^a^	Cannot read	1	0.2
	Can read parts of sentence	4	0.7
	Can read whole sentence	526	99.1
Employment^b^	Yes	365	68.6
	No	167	31.4
Travel time to facility (min)^c^	<15	40	7.5
	15–30	146	27.4
	30–60	211	39.7
	>60	135	25.4
Parity^d^ (*n* = 529)	Mean (SD)	1.1 (±0.8)	
	0	208	39.2
	1	183	34.5
	2 to 4	136	25.6
	≥5	4	0.8
Gestational age (weeks)^d^	28-31	245	46.1
	32-36	221	41.5
	≥37	66	12.4

Missing: ^a^2, ^b^1, ^c^1, ^d^2, and ^e^1.

**Table 2:** Clinical conditions of included women (antenatal care study population), *n* = 531.

Variable		ANC, *N* = 531	%
(a) Overall health rating	Very good	67	12.6
	Good	315	59.4
	Neither poor nor good	112	21.1
	Poor	29	5.5
	Very poor	7	1.3
(b) Have you been told you have anything wrong/any medical condition(s)?	No	180	34.0
	Yes	349	66.0
Are you taking any medication(s)?	No	201	37.9
	Yes	330	62.1
(c) Do you have any other medical conditions or other problem(s) you would like to report?	No	340	64.4
	Yes	188	35.6
(d) Obesity	BMI ≥ 30	269	51.0
Any preexisting conditions	Yes	258	48.6
	No	273	51.4
Leading direct preexisting conditions	Gestational diabetes	64	12.1
	Gestational hypertension	48	9.0
	Preeclampsia	17	3.2
	Urinary tract infection	11	2.1
	Pyelonephritis	5	0.9
	Others	8	1.5
Leading indirect preexisting conditions	Chronic hypertension	37	7.0
	Cervical insufficiency	16	3.0
	Preexisting diabetes mellitus	15	2.8
	Others	36	6.8
Symptoms	Mean SD	7.7 (± 5.6)	
(e) Any condition diagnosed on the day of the interview^∗^	No	472	90.9
	Yes	47	9.1
Number of conditions diagnosed^∗^, *N* = 454	0	11	2.4
	1	228	50.2
	2	92	20.3
	3	68	15.0
	≥4	55	12.1

Missing: (a) 1, (b) 2, (c) 3, (d) 4, and (e) 12. ^∗^Any of the conditions: clinical, WHODAS ≥ 37.4, anxiety score ≥ 10, depression score ≥ 10, exposure to domestic or sexual violence, sexual dissatisfaction, or substance use.

**Table 3:** Social and sexual conditions of the included women (antenatal care study population).

Variable		ANC, *N* = 531	%
(a) Substance use^∗^	No	461	87.3
	Yes	67	12.7
(b) Damage in the day to day due to substance use	No	48	77.4
	Yes	14	22.6
(c) Legal, social, or financial problems due to substance use	No	57	90.5
	Yes	6	9.5
(d) Family concern regarding substance use	No	21	33.3
	Yes	42	66.7
(e) Tried to stop but did not succeed	No	32	50.8
	Yes	31	49.2
Exposure to violence^∗∗^	No	495	93.2
	Yes	36	6.8
(f) Exposure to sexual violence^∗∗∗^	No	441	98.9
	Yes	5	1.1
	Refused to answer	2	0.4
Sex life during pregnancy	No	110	20.7
	Yes	421	79.3
(g) Reason for sexual abstinence	Does not have partner currently	24	22.0
	Medical restriction	43	39.4
	Partner does not want to	4	3.7
	She has little or no interest in sex	38	34.9
(h) Satisfaction with sex life	No	37	8.3
	Yes	411	91.7
Reason for sexual dissatisfaction	Little or no interest in sex	15	40.5
	Decreased genital sensation (feeling)	9	24.3
	Decreased vaginal lubrication (dryness)	10	27.0
	Problem reaching orgasm	8	21.6
	Pain during sex	21	56.8
	Refused to answer	1	2.7

Missing: (a) 3, (b) 5, (c) 4, (d) 4, (e) 4, (f) 83, (g) 1, and (h) 83. ^∗^Defined as use of the following substances: tobacco products, alcoholic beverages, marijuana (ganja), inhalants, sedatives or sleeping pills, hallucinogens, opioids, and/or any drugs by injection. ^∗∗^Women who responded no or never to the following question: (1) Are you afraid of your current/most recent husband or partner or anyone else? Would you say never, sometime, many times, most/all of the time? (2) Since pregnancy/delivery, was there ever a time when you were pushed, slapped, hit, kicked, or beaten by (any of) your husband/partner(s) or anyone else? ^∗∗∗^Women who responded no to the following question: (1) During this pregnancy, has your current husband/partner ever forced you to have sexual intercourse when you did not want to, for example, by threatening you or holding you down? (2) During this pregnancy, did you ever have sexual intercourse you did not want to because you were afraid of what your partner/husband might do if you refused? (3) During this pregnancy, did your husband/partner ever force you to do anything else sexual that you did not want or that you found degrading or humiliating?

**Table 4:** Mental and functional conditions of the study population.

Variable		ANC, *N* = 531	%
(a) Anxiety score	Mean (SD)	6.8 (±6.0)	
	Score ≥ 10	159	29.9
	Score < 10	372	70.1
(b) Depression score, *N* = 25	Mean (SD)	9.4 (±6.0)	
	Score ≥ 10	100	39.5
	Score < 10	153	60.5
(c) WHODAS score, *N* = 515	Mean (SD)	23.1 (±16.7)	
	Score < 37.4	410	79.6
	Score ≥ 37.4	105	20.4

(a) GAD-7: seven items, with four-point scale: 0 (not at all) to 3 (several days). A score ranging from 0 to 21 is considered a positive indicator of anxiety, equal to or greater than 10 [12]. (b) PHQ-9: nine items, with a four-point scale: 0 (not at all) to 3 (several days). A score ranging from 0 to 27 is considered a positive indicator of major depression, equal to or greater than 10 [13]. (c) WHODAS 12. Contains 12 items, the scores of each question were recoded and later the following formula was used [11]: Compute S1‐S12 = (S1 + S2 + S3 + S4 + S5 + S6 + S7 + S8 + S9 + S10 + S11 + S12)∗100/36.

**Table 5:** Factors associated with alterations in functionality (model 1), mental (model 2), and clinical alterations (model 3)—multivariate analysis.

Model/variable	PR	95%CI *p*/PR	*p*
(a) Model 1: functional impairment (*n* = 512)			
Overall health rating (neither poor nor good; poor; very poor)	3.37	2.41–4.71	<0.001
Literacy (can read parts of sentence or cannot read)	<0.01	<0.01–<0.01	<0.001
(b) Model 2: anxiety and depression (*n* = 284)			
Overall health rating (neither poor nor good; poor; very poor)	1.25	1.04–1.50	0.020
Literacy (can read parts of sentence or cannot read)	1.81	1.57–2.08	<0.001
(c) Model 3: clinical alterations (*n* = 522)			
Drug (yes)	0.70	0.54–0.91	0.007
^∗^BMI (≥30 kg/m^2^)	1.14	1.01–1.29	0.034

Multiple regression analysis by the Poisson regression model. ^∗^BMI: body mass index. (a) For model 1, the outcome was WHODAS ≥ 37.4, and predictors were the variables maternal age, marital status, education, literacy, employment, travel time to facility, parity, gestational age, BMI (≥30 kg/m^2^), overall health rating, any clinical condition, preexisting conditions, and taking any medication. (b) For model 2, the outcome was anxiety score ≥ 10 and depression score ≥ 10, and predictors were the variables maternal age, marital status, education, literacy, employment, travel time to facility, parity, gestational age, BMI (≥30 kg/m^2^), overall health rating, any clinical condition, preexisting conditions, and taking any medication. (c) For model 3, the outcome was any clinical condition reported by the woman, and predictors were the following variables: maternal age, marital status, education, literacy, employment, travel time to facility, parity, gestational age, BMI (≥30 kg/m^2^), alteration in mental health (anxiety score ≥ 10, depression score ≥ 10), sexual dissatisfaction, WHODAS ≥ 37.4, exposure to domestic or sexual violence, and substance use.

## Data Availability

Data used to support the findings of this study are available at reasonable request to the corresponding author. The WOICE tool is included as supplementary material.

## Supplementary Materials

Supplementary Materials Supplement 1: WHO-WOICE in English: maternal morbidity measurement tool (ANC) (version 2.0).
