# Supplementary material for: Overall Maternal Morbidity during Pregnancy Identified with the WHO-WOICE Instrument
Source: Biomed Res Int. 2020 Jul 17;2020:9740232. doi: 10.1155/2020/9740232 (PMC7382725; doi:10.1155/2020/9740232)
Supplement: Supplementary Materials — Supplement 1: WHO-WOICE in English: maternal morbidity measurement tool (ANC) (version 2.0). [file 9740232.f1.doc]

MATERNAL MORBIDITY MEASUREMENT TOOL – ANC (VERSION 2.0)

|  | **ANC SECTION 1: PATIENT HISTORY** | |
| --- | --- | --- |
|  | **Today's date** (yyyy/mm/dd) | **Hospital Registration Number: _____________________** |
| Q1 | **Interviewer Name: ______________________________** | |
|  | **Informed Consent.** *Please read the attached consent form to the patient. If the patient agrees to participate have her sign or fingerprint the form and take a picture of the signature/fingerprint with your tablet for documentation purposes. If the patient declines to participate, please attempt to ask her Q4-12.* | |
| Q2 | **Patient ID Number.** *ID number is located at the top right hand corner of the attached consent form.* | **#_______________________** |
|  | **Social & Demographic Information.** *Please read the following: "I would like to start by asking you some general questions about your life. If you don't understand a question or would like me to repeat it please feel free to stop and ask me."* | |
| Q3 | In what month and year were you born? *If unknown, please enter Jan 1950* | month: ________________ year:______________ |
| Q4 | How old were you on your last birthday? |  |
| Q5 | What is the highest level of school you attended? | □ none □ primary □ secondary □ higher |
| Q6 | What is your current marital status? *Please select one of the following choices*:  *(If single, ask: ever married?)* | Never married/Single _______ Currently married _______  Widowed _______ Separated _______ Divorced _______ Cohabiting _______ |
| Q7 | Have you worked in the last 12 months? | _______ No ________Yes |
| Q8 | Were you paid for this work? | _______ No ________Yes |
| Q9 | What district/county/parish/subnational level are you staying in? | Answers vary per site |
| Q10 | How long did it take you to get from your house to your health facility today? | □ <15 mins □ 15-30 mins □ 30 mins - 1hr □ >1hr |
| Q11 | Now I would like you to read this sentence to me*:*  "The child is reading a book."  IF RESPONDENT CANNOT READ WHOLE SENTENCE, PROBE:  Can you read any part of the sentence to me? | ____ cannot read at all ____ able to read only parts of the sentence ____ able to read whole sentence ____ blind/visually impaired |

|  | **Obstetric History.** *Please read the following: "We are working on a project to get a better idea of how women feel throughout and after their pregnancies in order to improve what we know and how we can better serve you and other pregnant women in the future. Now I would like to ask you some questions about other times you have been pregnant, if any. Again, please ask me if you don't understand the question."* | | | | | | | | | | | | | | | |
| --- | --- | --- | --- | --- | --- | --- | --- | --- | --- | --- | --- | --- | --- | --- | --- | --- |
| Q12 | How many babies have you given birth to (that lived or died) after 22 weeks or 5 months of pregnancy? | | | | | | | | | |  | | | | | |
| Q13 | How many babies did you lose before 22 weeks or 5 months of pregnancy? | | | | | | | | | |  | | | | | |
| Q14 | How many children have you given birth to that are now alive? | | | | | | | | | |  | | | | | |
| Q15 | How many times have you been pregnant? (including this pregnancy, and times when you did not give birth) | | | | | | | | | |  | | | | | |
| A-1 | Are you currently pregnant with more than one baby? | | | | | | | | | | _______No _______Yes □ Don't know | | | | | |
| A-2 | What is your estimated date of delivery (EDD)? (day/month/year) (If woman does not know, please check records) | | | | | | | | | | (dd/mm/yyyy) ____________ □ Don't know | | | | | |
|  | **Most Recent Pregnancy/Delivery.** *Please read the following: "Now I would like to ask you some more intimate questions, if you do not feel comfortable answering them please let me know at any time."* | | | | | | | | | | | | | | | |
| A-3 | Since you became pregnant, have you continued having sex? | | | | _______ No ________Yes (Please skip to Q16) | | | | | | | | | | | |
| A-4 | If not, why not? | | | | 1) Does not have partner currently  2) Dr says not to  3) Partner does not want to  4) She has little or no interest in sex | | | | | | | | | | | |
| Q16 | Since you became pregnant, are you satisfied with your sex life? | | | | _______ No ________Yes (Please skip to Q19) | | | | | | | | | | | |
| Q17 | Since you became pregnant, the problem(s) with your sex life is: *(Please select all the choices that apply)* | | | | 1) Problem with little or no interest in sex ______ 2) Problem with decreased genital sensation (feeling) _______ 3) Problem with decreased vaginal lubrication (dryness) ______ 4) Problem reaching orgasm ______ 5) Problem with pain during sex _______ 6) Refused to answer | | | | | | | | | | | |
| Q18 | *If more than one option is chosen for Q17, then please ask the patient* "Which problem is the most bothersome?" *and circle the corresponding answer.* | | | | | | | | | | | | | | | |
|  | **Risk factors/Environment.** *Please read the following: "The next few questions I will ask may be a bit difficult, so please feel free to ask for a break or stop at any time. I want to remind you that this is confidential and no one will know how you answered. Also, if after this section you'd like to talk more about the questions, I will give you information on where to seek help. We are asking these questions to better understand your health situation and those of other women who might have similar experiences in the future." If the patient does not want to answer, you can skip the remaining questions and offer her access to the services available (see note at the end of the page).* | | | | | | | | | | | | | | | |
| Q19 | During this pregnancy, have you used any of the following substances: tobacco products, alcoholic beverages, cannabis, inhalants for non-medical use? | | | | | | | | | | | _______No _______Yes | | | | |
| Q20 | During this pregnancy, have you used any substances: sedatives or sleeping pills, hallucinogens, opioids, and/or any drugs by injection, etc., for non-medical use? | | | | | | | | | | | _______No (or No on Q19, Please skip to Q25)  _______Yes | | | | |
|  | *If she answers yes to Q19 or Q20 please ask the remaining questions:* | | | | | | | | | | | | | | | |
| Q21 | During this pregnancy, have you failed to do what was normally expected of you because of your consumption of any of the abovementioned substances? | | | | | | | | | | | _______No _______Yes | | | | |
| Q22 | During this pregnancy, has your use of any of the aforementioned substances led to health, social, legal, or financial problems? | | | | | | | | | | | _______No _______Yes | | | | |
| Q23 | During this pregnancy, has a friend or relative or anyone else *ever* expressed concern about your use of any substance? | | | | | | | | | | | _______No _______Yes | | | | |
| Q24 | During this pregnancy, have you ever tried to cut down on using any substance, but failed? | | | | | | | | | | | _______No _______Yes | | | | |
|  | **Violence.** *Please read the following: "The next few questions I will ask may also be a bit difficult, so please feel free to ask for a break or stop at any time. I want to remind you that this is confidential and no one will know how you answered. Also, if after this section you'd like to talk more about the questions, I will give you information on where to seek help. We are asking these questions to better understand your health situation and those of other women who might have similar experiences in the future."* | | | | | | | | | | | | | | | |
| Q25 | Are you afraid of your current/most recent husband or partner or anyone else? Would you say…? | | | | | | _______Never _______Sometimes _______ Many times _______ Most/all of the times _______ Don't know/Don't remember _______ Refused/No answer | | | | | | | | | |
| Q26 | During this pregnancy, was there ever a time when you were pushed, slapped, hit, kicked or beaten by (any of) your husband/partner(s) or anyone else? | | | | | | _______No  _______Yes  _______ Don't know/Don't remember _______ Refused/No answer | | | | | | | | | |
|  | *If she answers yes (any response other than never or no) to Q25 or Q26 please ask the remaining questions:* | | | | | | | | | | | | | | | |
| Q27 | During this pregnancy, has your current husband/partner ever forced you to have sexual intercourse when you did not want to, for example by threatening you or holding you down? IF NECESSARY: We define sexual intercourse as vaginal, oral or anal penetration. | | | | | | _______No _______Yes  _______ Don't know/Don't remember _______ Refused/No answer (Skip to instructions) | | | | | | | | | |
| Q28 | During this pregnancy, did you ever have sexual intercourse you did not want to because you were afraid of what your partner/husband might do if you refused? | | | | | | _______No _______Yes  _______ Don't know/Don't remember _______ Refused/No answer (Skip to instructions) | | | | | | | | | |
| Q29 | During this pregnancy, did your husband/partner ever force you to do anything else sexual that you did not want or that you found degrading or humiliating? | | | | | | _______No _______Yes  _______ Don't know/Don't remember _______ Refused/No answer (Read the instructions) | | | | | | | | | |
|  | **End of Module 1: Patient History Thank you for answering the questions, we will now move on to the 2nd module of the questionnaire.** | | | | | | | | | | | | | | | |
|  | | **ANC SECTION 2: PATIENT SYMPTOMS** | | | | | | | | | | | | | | |
|  | | **WHODAS.** *Please read the following: "Now, I would like to ask you some more questions about your everyday activities. This part of the interview is about difficulties people have because of health conditions. (Hand flashcard #1 to respondent) By health condition I mean diseases or illness, or other health problems that may be short or long lasting; injuries; mental or emotional problems; and problems with alcohol or drugs. Remember to keep all of your health problems in mind as you answer the questions.   When I ask you about difficulties in doing an activity think about...(Point to flashcard #1):   - increased effort  - discomfort or pain  - slowness  - changes in the way you do the activity  When answering, I'd like you to think back over the past 30 days. I would also like you to answer these questions thinking about how much difficulty you have had, on average, over the past 30 days, while doing the activity as you usually do it. (Hand flashcard #2 to respondent)   Use this scale when responding. (Read scale aloud): None, mild, moderate, severe, extreme or cannot do. (Ensure that the respondent can easily see flashcards #1 and #2 throughout the interview. Please continue to next question...)"* | | | | | | | | | | | | | | |
|  | | **In the past 30 days, how much difficulty did you have in:** | | | | **None** | | | **Mild** | | | | **Moderate** | **Severe** | **Extreme or  cannot do** | |
| Q30 | | Standing for long periods such as 30 minutes? | | | | 1 | | | 2 | | | | 3 | 4 | 5 | |
| Q31 | | Taking care of your household responsibilities? | | | | 1 | | | 2 | | | | 3 | 4 | 5 | |
| Q32 | | Learning a new task, for example, learning how to get to a new place? | | | | 1 | | | 2 | | | | 3 | 4 | 5 | |
| Q33 | | How much of a problem did you have joining in community activities (for example, festivities, religious or other activities) in the same way as anyone else can? | | | | 1 | | | 2 | | | | 3 | 4 | 5 | |
| Q34 | | How much have you been emotionally affected by your health problems? | | | | 1 | | | 2 | | | | 3 | 4 | 5 | |
|  | | **In the past 30 days, how much difficulty did you have in:** | | | | **None** | | | **Mild** | | | | **Moderate** | **Severe** | **Extreme or cannot do** | |
| Q35 | | Concentrating on doing something for ten minutes? | | | | 1 | | | 2 | | | | 3 | 4 | 5 | |
| Q36 | | Walking a long distance such as a kilometre [or equivalent]? | | | | 1 | | | 2 | | | | 3 | 4 | 5 | |
| Q37 | | Washing your whole body? | | | | 1 | | | 2 | | | | 3 | 4 | 5 | |
| Q38 | | Getting dressed? | | | | 1 | | | 2 | | | | 3 | 4 | 5 | |
| Q39 | | Dealing with people you do not know? | | | | 1 | | | 2 | | | | 3 | 4 | 5 | |
| Q40 | | Maintaining a friendship? | | | | 1 | | | 2 | | | | 3 | 4 | 5 | |
| Q41 | | Your day-to-day work/school? | | | | 1 | | | 2 | | | | 3 | 4 | 5 | |
| Q42 | | Overall, in the past 30 days, how many days were these difficulties present? | | | | | | | | | | | | Record number of days__ | | |
| Q43 | | In the past 30 days, for how many days were you totally unable to carry out your usual activities or work because of any health condition? | | | | | | | | | | | | Record number of days__ | | |
| Q44 | | In the past 30 days, not counting the days that you were totally unable, for how many days did you cut back or reduce your usual activities or work because of any health condition? | | | | | | | | | | | | Record number of days__ | | |
| Q45 | | In the past 30 days, how would you rate your overall health? | | | | 1 | | | 2 | | | | 3 | 4 | | 5 |
|  |  |  |  |  |  | Very Good | | | Good | | | | Neither poor nor good | Poor | | Very poor |
|  | | **General Symptom(s).**  *Please read the following: "The next few questions I will ask about how you have been feeling, physically, during this pregnancy. Feel free to ask for a break or stop at any time."* | | | | | | | | | | | | | | |
|  | | **In the last 2 weeks, have you experienced any of the following:** *(Please check box if yes. If no, skip to next symptom)* | | | | | | | | | | | | | | |
| Q46 | | □ chills | □ nausea | | | | | □ fever | | | | | | | | |
|  | | □ headache | □ light-headedness | | | | |  | | | | | | | | |
|  | |  | | | | | | | | | | | | | | |
| Q47 | | □ stiff neck | □ muscle spasms | | | | | □ tremor | | | | | | | | |
|  | | □ sweating profusely/night sweats, unrelated to the heat (diaphoresis) | | | | | | | | | | | | | | |
|  | |  | | | | | | | | | | | | | | |
| Q48 | | □ chest pain | □ decreased exercise tolerance or fatigue | | | | | □ heart beating very fast/too fast (palpitations) | | | | | | | | |
|  | | □ seeing stars or spots, blurry vision/flashing lights/floaters or having visual disturbance or visual loss | | | | | | | | | | | | | | |
|  | |  | | | | | | | | | | | | | | |
| Q49 | | □ red/inflamed or bleeding gums | □ cough | | | | | □ difficulty breathing | | | | | | | | |
|  | | □ breathing faster than usual |  | | | | |  | | | | | | | | |
|  | |  | | | | | | | | | | | | | | |
| Q50 | | □ vomiting | □ vomiting with blood | | | | | □ abdominal discomfort or pain | | | | | | | | |
|  | | □ changes in appetite or eating habits |  | | | | |  | | | | | | | | |
|  | |  | | | | | | | | | | | | | | |
| Q51 | | □ pain during urination (dysuria) | □ abnormal urination | | | | | □ changes in bowel habits | | | | | | | | |
|  | | □ rectal pressure/pain |  | | | | |  | | | | | | | | |
|  | |  | | | | | | | | | | | | | | |
| Q52 | | □ skin rash or lesion | □ itching (pruritus) | | | | | □ breast tenderness or redness, or feel breast lump (mass) or swelling | | | | | | | | |
|  | |  | | | | | | | | | | | | | | |
| Q53 | | □ arthralgia/arthritis (joint pain) | □ tenderness in leg or calf | | | | | □ sudden swelling in leg(s) or calf(-ves) | | | | | | | | |
|  | | □ back pain |  | | | | |  | | | | | | | | |
|  | |  | | | | | | | | | | | | | | |
| Q54 | | □ vaginal bleeding (after sex) | □ painful intercourse (dyspareunia) | | | | | □ pelvic pain | | | | | | | | |
|  | | □ vaginal discharge (abnormal in color and/or smell) | □ spotting or light vaginal bleeding | | | | |  | | | | | | | | |
|  | |  | | | | | | | | | | | | | | |
| Q55 | | **Since you became pregnant, have you EVER experienced any of the following:** | | **Q56 Since you became pregnant, have you EVER experienced any of the following:** | | | | | | | | | | | | |
|  | | □ urinating blood | | □ stroke | | | | | | | | | | | | |
|  | | □ night blindness (difficulty seeing in the dark) | | □ hemorrhoids/piles | | | | | | | | | | | | |
|  | | □ unintentional weight loss | | □ loss of teeth | | | | | | | | | | | | |
|  | | □ gained too much weight (excessive weight gain: >1kg per week) | | □ swollen hands | | | | | | | | | | | | |
|  | |  | | □ seizure/fit | | | | | | | | | | | | |
| Q57 | | **Since you became pregnant, have you been told you have anything wrong/any medical condition?** | | | | | | | | _______No (Skip to Q59) _______Yes | | | | | | |
| Q58 | | *If yes, please specify:* | | | | | | | | | | | | | | |
|  | |  | | | | | | | | | | | | | | |
|  | |  | | | | | | | | | | | | | | |
| Q59 | | **Do you have any other medical conditions or problems you would like to report?** | | | | | | | | _______No (Skip to Q61) _______Yes | | | | | | |
| Q60 | | *If yes, please specify:* | | | | | | | | | | | | | | |
|  | |  | | | | | | | | | | | | | | |
|  | |  | | | | | | | | | | | | | | |

|  | **Mental Health.** *Please read the following: "The next few questions I will ask about how you have been feeling/your mood during this pregnancy, feel free to ask for a break or stop at any time. I want to remind you that this is confidential and no one will know how you answered. Also, if after this section you'd like to talk more about the questions, I will give you information on where to seek help***."** Present card # 4 and give a score of 0 to 3 | | | | |
| --- | --- | --- | --- | --- | --- |
|  | Over the last 2 weeks, how often have you been bothered by the following problems? | **Not at all** | **Several days** | **More than half the days** | **Nearly every day** |
| Q61 | Feeling nervous, anxious or on edge | 0 | 1 | 2 | 3 |
| Q62 | Not being able to stop or control worrying | 0 | 1 | 2 | 3 |
| Q63 | Worrying too much about different things | 0 | 1 | 2 | 3 |
| Q64 | Trouble relaxing | 0 | 1 | 2 | 3 |
| Q65 | Being so restless that it is hard to sit still | 0 | 1 | 2 | 3 |
| Q66 | Becoming easily annoyed or irritable | 0 | 1 | 2 | 3 |
| Q67 | Feeling afraid as if something awful might happen | 0 | 1 | 2 | 3 |
|  | Over the past 2 weeks, how often have you been bothered by any of the following problems? | **Not at all** | **Several days** | **More than half the days** | **Nearly every day** |
| Q68 | Little interest or pleasure in doing things | 0 | 1 | 2 | 3 |
| Q69 | Feeling down, depressed or hopeless | 0 | 1 | 2 | 3 |
|  | *If she scores greater than 0 to Q68 or Q69, please ask the remaining questions:* |  |  |  |  |
| Q70 | Trouble falling asleep, staying asleep or sleeping too much | 0 | 1 | 2 | 3 |
| Q71 | Feeling tired or having little energy | 0 | 1 | 2 | 3 |
| Q72 | Poor appetite or overeating | 0 | 1 | 2 | 3 |
| Q73 | Feeling bad about yourself - or that you're a failure or have let yourself or your family down | 0 | 1 | 2 | 3 |
| Q74 | Trouble concentrating on things, such as reading the newspaper or watching television | 0 | 1 | 2 | 3 |
| Q75 | Moving or speaking so slowly that other people could have noticed. Or, the opposite, being so fidgety or restless that you have been moving around a lot more than usual | 0 | 1 | 2 | 3 |
| Q76 | Thoughts that you would be better off dead or of hurting yourself in some way | 0 | 1 | 2 | 3 |
|  | **Please add up all the points for Q61-Q67. Please add up all the points for Q68-Q76. If total score on EITHER set of questions is equal to 10 or higher, please refer the patients to mental health service.** | | | | |
|  | **End of Module 2: Symptoms Thank you for answering the questions, we will now move on to the 3rd and final module of the questionnaire, the physical exam.  Do you have any questions?** | | | | |

|  | **ANC SECTION 3: SIGNS/PHYSICAL EXAM** | |
| --- | --- | --- |
|  | **General physical exam.** *Data collected in medical records on physical examination if it is routine.* | |
| Q77 | Body weight today: | ______ kg |
| Q78 | Height: | ______ cm |
| Q79 | Body temperature: | ______ °C |
| Q79a | Where on the body was the temperature taken? | □ oral □ axillary  □ other (please specify):__________ |
| Q80 | Pulse rate: | ____/min |
| Q81 | Respiratory rate: | ____/min |
| Q82 | Resting Systolic BP |  |
| Q83 | Resting Diastolic BP |  |
| Q85 | Does the woman present with any pre-existing conditions? | |
|  | □ No | □ Syphilis |
|  | □ HIV | □ STI (syndromic) |
|  | □ Chronic Hypertension | □ Hepatitis B |
|  | □ Gestational hypertension | □ Trichomoniasis |
|  | □ Diabetes | □ Malaria |
|  | □ Gestational diabetes | □ Typhoid Fever |
|  | □ UTI | □ Pityriasis |
|  | □ Pyelonephritis | □ Dermatomycosis |
|  | □ Varicose veins | □ Oral candida |
|  | □ Pre-eclampsia | □ Pelvic Candida |
|  | □ Premature preterm rupture of membranes | □ Pelvic Inflammatory Disease |
|  | □ Premature Rupture of membranes in the term | □ Vaginitis |
|  | □ Placenta Previa | □ Cervicitis |
|  | □ Cervical Insufficiency | □ Bacterial vaginosis |
|  | □ Cervical dysplasia | □ Anogenital warts |
|  | □ Olygoamnio | □ Chickungunya |
|  | □ Fibroids | □ Herpes |
|  | □ Small for gestational age | □ Sickle cell anemia |
|  | □ Ante-partum Hemorrhage |  |
|  | □ Sepsis |  |
|  | □ Surgical wound infection |  |
|  | □ Tuberculosis |  |
|  | □ other (please specify): ______________________________________________________________________ | |
| Q86 | Is the woman currently on any medications? |  |
|  | □ No | □ Analgesic |
|  | □ Antifungal (not vaginal) | □ Antiepileptic |
|  | □ Antimalarial | □ Antiretroviral |
|  | □ Vitamins and minerals | □ Antihypertensive |
|  | □ Hematinic | □ Diuretic |
|  | □ Antibiotics | □ Magnesium Sulfate |
|  | □ Antifungal (vaginal) | □ Anticoagulant |
|  | □ Thyroid medication | □ Antenatal corticoid |
|  | □ Antiparasitic | □ Asthma medications |
|  | □ Metronidazole | □ Topic corticoid |
|  | □ Anti-Bv / Tric | □ Topic Antibiotics |
|  | □ Antiviral (not HIV) | □ Anti-cough medication |
|  | □ Tuberculosis treatment | □hemorrhoid medication |
|  | □ Antiemetic | □ Antihistamine |
| Q143 | When present, please mark the following diagram with the letters corresponding to the questions above:- R for rash(es)- L for lesion(s)- B for bruises- SH for self-harm- DV for domestic violence |  |
| Q87 | Does she present with any of the following? *(choose all that apply)* | |
|  | □ Pitting ankle oedema | □ leg swelling |
|  | □ Pitting lower back oedema | □ calf tenderness |
|  | □ Oedema of the hands and feet | □ none |
|  | **Obstetric exam - to be completed always** | |
| A-5 | Please check symphysis-fundal height | _______ cm |
| A-6 | Please check the fetal heart rate | _____/min |
| A-7 | What is the presentation of the fetus? | □ Cephalic □ Breech  □ Transverse □ >1 fetus |
|  | **Breast exam**  **Triggered only if woman reports the following breast related symptoms:**  **-** breast tenderness or redness, or feel breast lump (mass) or swelling  - ??? | |
| Q91 | Does she present with cracked nipple(s)? | _______No _______Yes |
| Q92 | Does she present with engorged breast(s)? | _______No _______Yes |
| Q93 | Does she present with localized breast tenderness? | _______No _______Yes (If yes, please mark where in the diagram below) |
|  |  | **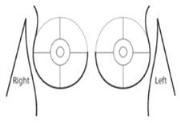** |
| Q94 | Does she present with breast abscess(es)? | _______No _______Yes (If yes, please mark where in the diagram below) |
|  |  | **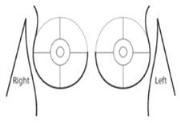** |
| Q95 | Does she present with palpable breast lump(s)? | _______No _______Yes (If yes, please mark where in the diagram below) |
|  |  | 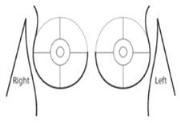 |

|  | **Abdominal exam**  **Triggered only if woman reports the following symptoms:**  **-** vomiting (with or without blood)  - abdominal discomfort or pain  - changes in appetite or eating habits  - ??? | |
| --- | --- | --- |
| Q96 | Does she present with abdominal tenderness? | _______No _______Yes (If yes, please mark where in the diagram below) |
|  |  | Left  Lower Quadrant  Right Lower Quadrant    Left Upper Quadrant    Right Upper Quadrant |
| Q97 | Does she present with abdominal masses? | _______No _______Yes (If yes, please mark where in the diagram below) |
|  |  | Left  Lower Quadrant    Right Lower Quadrant    Left  Upper Quadrant    Right Upper Quadrant |
| Q98 | Does she have a C-section scar? | _______No _______Yes |

|  | **Pelvic exam**  **Triggered only if suspected infection or labor/rapture of membranes or if woman reports the following symptoms:**  **-** vaginal bleeding (after sex)  - painful intercourse (dyspareunia)  - pelvic pain  - vaginal discharge (abnormal in color and/or smell)  - spotting or light vaginal bleeding  - ??? | | |
| --- | --- | --- | --- |
| Q164 | Does she present with any of the following types of FGM? *(please check one)* | □ None | |
|  |  | 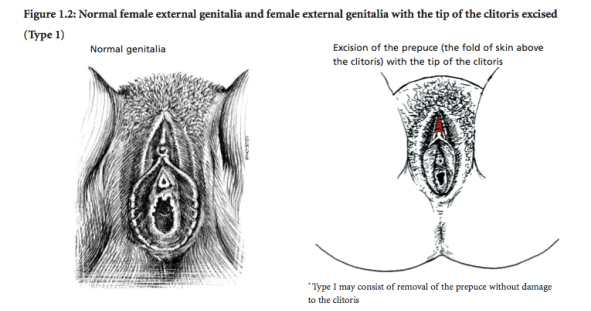□ Type 1: Partial or total removal of the clitoris and/or the prepuce (clitoridectomy) | |
|  |  | 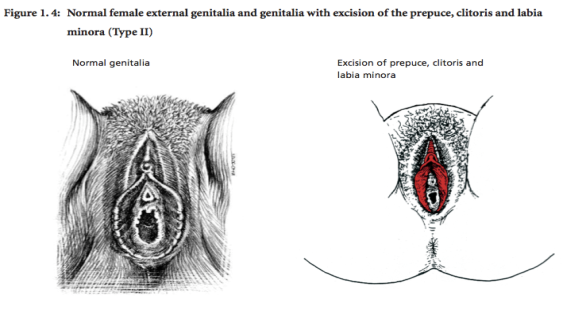□ Type 2: Partial or total removal of the clitoris and the labia minora, with or without excision of the labia majora (excision) | |
|  |  | 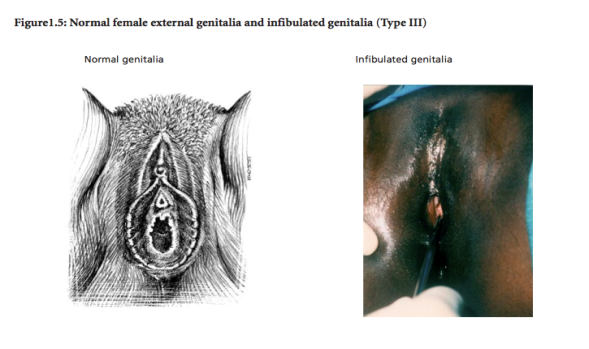□ Type 3: Narrowing of the vaginal orifice with the creation of a covering seal by cutting and appositioning the labia minora and/or the labia majora, with or without excision of the clitoris (infibulation) | |
|  |  | 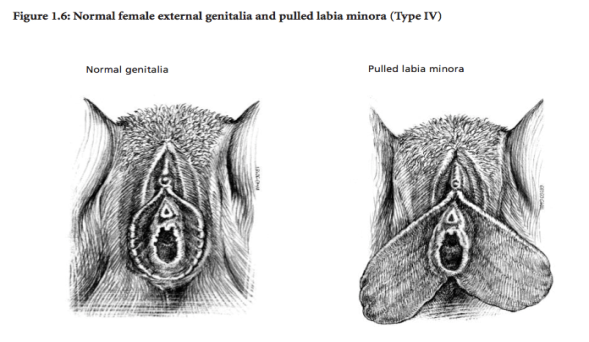□ Type 4: All other harmful procedures to the female genitalia for non-medical purposes, for example: pricking, pulling, piercing, incising, scraping and cauterization | |
|  | *Vulva, Vagina & Perineum* | | |
| Q99 | Does she present with any of the following in the vulva? (Please check all that apply) | | |
|  | □ leakage of urine | | □ excoriation |
|  | □ labial swelling | | □ none |
| Q100 | Does she present with any of the following in the vagina? (Please check all that apply) | | |
|  | □ lesion(s) | | □ defects |
|  | □ none | |  |
| Q101 | Does she present with any of the following in the perineum? (Please check all that apply) | | |
|  | □ excoriation | | □ tear |
|  | □ swelling | | □ none |
| A-8 | Are there signs of premature membrane rupture? | | _______No _______Yes _______Uncertain |
| Q102 | Does she present with any abnormal vaginal discharge? | | _______No _______Yes |
| Q103 | If yes, please explain colour: _____________________________________________________________________ | | |
|  | **Records & Tests** | | |
|  | **INVESTIGATIONS - Routine Tests.** *Please look through the patient's most up to date medical record to answer the following questions regarding the lab tests she had done.* | | |
| Q104 | hemoglobin (hemocue) | | _______ No _______Yes  _______ Yes, but results not available If yes, please write in the results: ______ Hb(g/dl) |
| A-9 | syphilis (VDRL) | | _______ No _______Yes  _______ Yes, but results not available If yes, please mark one of the results: ____ Reactive _____ Non-reactive _____ Don't know |
| Q105 | nitrite (dipstick) | | _______No _______Yes *If yes, please write in the results:*  ________ + ________ ++  ________ +++ _______ None |
| Q106 | leucocytes (dipstick) | | _______No _______Yes *If yes, please write in the results:*  ________ + ________ ++  ________ +++ _______ None |

| A-10 | ultrasound (obstetric only - where available) | _______No _______Yes  *If yes, please write in the results:*  _________________________________  _________________________________  **Inform: gestational age, presentation, estimated fetal weight, ILA and Doppler** |
| --- | --- | --- |
| Q107 | HIV | _______ No _______Yes _______ Yes, but results not availableIf yes, please mark one of the results:____ Negative _____ Positive _____ Inconclusive |
|  | **INVESTIGATIONS - Selective Tests.** *Please look through the patient's most up to date medical record to answer the following questions regarding the lab tests she had done.* | |
| Q108 | glucometer (random blood sugar) | _______ No _______Yes  _______ Yes, but results not available If yes, please write in the results: _________ mMol/L- mg / dl |
|  | ***[Include other tests relevant to setting here]*** |  |
| Q109 | **Did you or the nurse midwife prescribe/refer the woman to buy any medication today?** | _______No _______Yes |
|  | *If yes, please specify the medications(s):* | |
|  |  | |
| Q110 | **Did you or the nurse midwife diagnose the patient with any condition(s) today?** | _______No _______Yes |
|  | *If yes, please specify the condition(s):* | |
|  |  | |
| Q111 | **Do you or the nurse midwife have any other comments/notes on the patient?** | _______No _______Yes |
|  | *If yes, please specify:* | |
| Q112 | Did the woman need some kind of referral? | _______No _______Yes |
|  | *If yes, please specify the referral* | Psychological support ___  Medical care ____  Social service attendance ____ |
|  | **End of Module 3: Patient Signs Thank you for participating in this survey, we have come to the end of the questionnaire.  Do you have any questions for me?** | |
|  |

**Flashcards**

*Flashcard #1*

THE CHILD IS READING A BOOK

*Flashcard #2*

Health conditions:

- diseases, illnesses or other health problems

- mental or emotional problems

- problems with alcohol

- problems with drugs

Having difficulty with an activity means:

- increased effort

- discomfort or pain

- slowness

- changes in the way you do the activity

Think about the past 30 days only.

*Flashcard #3*

5 Extreme or cannot do

4 Severe

3 Moderate

2 Mild

1 None

*Flashcard #4*

3 Nearly every day

2 More than half the days

1 Several days

0 Not at all
